# CARD11 is a prognostic biomarker and correlated with immune infiltrates in uveal melanoma

**DOI:** 10.1371/journal.pone.0255293

**Published:** 2021-08-09

**Authors:** Xueying Shi, Shilin Xia, Yingming Chu, Nan Yang, Jingyuan Zheng, Qianyi Chen, Zeng Fen, Yuankuan Jiang, Shifeng Fang, Jingrong Lin

**Affiliations:** 1 Clinical Laboratory of Integrative Medicine, The First Affiliated Hospital of Dalian Medical University, Dalian, Liaoning, China; 2 Institute (College) of Integrative Medicine, Dalian Medical University, Dalian, Liaoning, China; 3 Department of Integrated Traditional Chinese Medicine, Peking University First Hospital, Beijing, China; 4 Department of Nephrology, The First Affiliated Hospital of Dalian Medical University, Dalian, Liaoning, China; 5 Department of Dermatology, The First Affiliated Hospital of Dalian Medical University, Dalian, Liaoning, China; 6 Department of Ophthalmology, The First Affiliated Hospital of Dalian Medical University, Dalian, Liaoning, China; University of Queensland Diamantina Institute, AUSTRALIA

## Abstract

Uveal melanoma (UVM), the most common primary intraocular malignancy, has a high mortality because of a high propensity to metastasize. Our study analyzed prognostic value and immune-related characteristics of *CARD11* in UVM, hoping to provide a potential management and research direction. The RNA-sequence data of 80 UVM patients were downloaded from The Cancer Genome Atlas database and divided them into high- and low-expression groups. We analyzed the differentially expressed genes, enrichment analyses and the infiltration of immune cells using the R package and Gene-Set Enrichment Analysis. A clinical prediction nomogram and protein-protein interaction network were constructed and the first 8 genes were considered as the hub-genes. Finally, we constructed a competing endogenous RNA (ceRNA) network by Cytoscape and analyzed the statistical data via the R software. Here we found that *CARD11* expression had notable correlation with UVM clinicopathological features, which was also an independent predictor for overall survival (OS). Intriguingly, *CARD11* had a positively correlation to autophagy, cellular senescence and apoptosis. Infiltration of monocytes was significantly higher in low *CARD11* expression group, and infiltration of T cells regulatory was lower in the same group. Functional enrichment analyses revealed that *CARD11* was positively related to T cell activation pathways and cell adhesion molecules. The expressions of hub-genes were all increased in the high *CARD11* expression group and the ceRNA network showed the interaction among mRNA, miRNA and lncRNA. These findings show that high *CARD11* expression in UVM is associated with poor OS, indicating that *CARD11* may serve as a potential biomarker for the diagnosis and prognosis of the UVM.

## 1. Introduction

Uveal melanoma (UVM), with a high mortality because of a high propensity to metastasize and recur, is well known as the most common primary intraocular malignancy in adults. It arises from intraocular melanocytes of the choroid, the ciliary body, and the iris [1]. The eye is considered as an immune-privileged organ because immune responses are controlled in order to protect eyesight, helping UVM cells evading immune surveillance and providing immune evasion advantage for UVM [2]. Primary uveal melanoma can be cured by surgery or radiotherapy. However, the following problem of these treatments is vision loss, which is a worrisome adverse effect [3]. Approximately half of UVM patients will have further deterioration, of whom the survival rate remains bleak. Chemotherapies and directed treatments do not usually maintain long-term tumor control. Therefore, immunotherapy is currently a promising therapy option [4]. At the meanwhile, it is necessary to explore novel prognostic biomarkers or efficacious therapeutic targets.

Caspase Recruitment Domain Family Member 11 (*CARD11*) is a multi-domain scaffold protein, carrying a characteristic caspase-associated recruitment domain. It belongs to not only the CARD protein family but also the membrane-associated guanylate kinase family, a class of proteins that act as molecular scaffolds [5, 6]. *CARD11* serves as a crucial bridge between antigen recognition and the activation of downstream nuclear factor κB (NF-κB) in lymphocytes, and is an essential signal center in adaptive immune response [7]. *CARD11* gain-of-function mutation can stimulate constitutive NF-κB activity and induce proliferation of invasive B-cell lymphocytes, contributing to diffuse large B-cell lymphoma and other lymphatic carcinomas [5, 8–11]. *CARD11* depletion can have an anti-tumor effect by inhibiting the systemic autoimmunity [12]. Positive *CARD11* expression is associated with an inferior event free survival [11]. Besides, it is reported that *CARD11* is also related to adult T cell leukemia/lymphoma and colorectal cancer [13, 14].

In this study, we aimed to demonstrate the correlation between the profile of *CARD11* in UVM, and data of mRNA and lncRNA were obtained from The Cancer Genome Atlas (TCGA) and Gene Expression Omnibus (GEO) databases. We systematically integrated and analyzed around the expression of *CARD11* based on bioinformatic and statistical methods including differentially expressed genes (DEGs) analysis, Gene Ontology (GO), Kyoto Encyclopedia of Genes and Genomes (KEGG) pathway and Gene-Set Enrichment Analysis (GSEA) analyses. To gain a more in-depth understanding of the relationship between the expression of the *CARD11* with the clinical features and with the immune infiltration level in UVM, Nomogram and Cox regression analysis and immune cell infiltration analysis were carried. In addition, we used these DEGs to construct a Competing endogenous RNA (ceRNA) network. The present study showed that increased *CARD11* was related to poor prognosis and can be considered a potential biomarker of UVM, which suggested its likely function as a marker for clinical diagnosis or even novel therapeutic target in patients with UVM.

## 2. Materials and methods

### 2.1. Data source

The RNA sequencing gene expression data (FPKM value) of 80 patients with UVM were downloaded from TCGA GDC website (https://portal.gdc.cancer.gov/) and divided into profiles of mRNA and lncRNA expression. The expression values were transformed by log2 (FPKM+1) to reveal differential level of *CARD11* between normal and tumor tissue. UCSC Xena (http://xena.ucsc.edu/) was performed to provide the clinicopathological features and prognosis of the UVM patients, including gender, age and stage. The detailed clinical information of patients with UVM is shown in Table 1.

**Table 1:** Characteristics and clinical data of uveal melanoma patients from TCGA.

Variables	All patients (n = 80)	Low expression (n = 40)	High expression (n = 40)	*P value*
Gender				0.499
Female	35 (43.8%)	19 (47.5%)	16 (40.0%)
Male	45 (56.2%)	21 (52.5%)	24 (60.0%)
Age				0.369
<60	36 (45.0%)	20 (50.0%)	16 (40.0%)
≥60	44 (55.0%)	20 (50.0%)	24 (60.0%)
Pathologic stage				0.04*
II	36 (45.0%)	24 (60.0%)	12 (30.0%)
III	40 (50.0%)	16 (40.0%)	24 (60.0%)
IV	4 (5.0%)	0 (0.0%)	4 (10.0%)
Histological type		<0.001***		
Epithelioid Cell	13 (16.3%)	3 (7.5%)	10 (25.0%)
Mixed	37 (46.2%)	13 (32.5%)	24 (60.0%)
Spindle Cell	30 (37.5%)	24 (60.0%)	6 (15.0%)
Type				<0.001***
metastatic	19 (23.8%)	1 (2.5%)	18 (45.0%)
non-metastatic	61 (76.3%)	39 (97.5%)	22 (55.0%)

The gene expression data of GSE22138 and the clinicopathological features of patients were downloaded from the GEO database as verification dataset [15]. The type of samples was Homo sapiens, and the platform was based on GPL570 [HG-U133_Plus_2] Affymetrix Human Genome U133 Plus 2.0 Array, including 63 UVM tissue samples.

### 2.2. Differentially expressed gene analysis

To investigate prognostic value of *CARD11* in UVM patients, 80 UVM samples were divided into two groups of 40 samples by the best cut-off point: high *CARD11* expression and low *CARD11* expression. Based on the FPKM value, the patients were stratified into high-level group and low-level group according to median expression of *CARD11*. Additionally, the count value of the patient was downloaded followed by a standardization with Limma-voom, then we further analyzed a differential expression. [16]. |log FC|>1.5 and adjusted *P* <0.05 were considered as threshold values for the DEGS. The differential analysis was presented as heatmaps and volcano plots.

### 2.3. Gene-set enrichment analysis

GO analysis was used in large-scale functional enrichment research, including biological process, molecular function and cellular component. KEGG is a widely used database storing information related to genome, biological pathways, diseases, and drugs. ClusterProfiler R package was used to annotate GO information and enrich KEGG pathway [17]. The FDR score less than 0.05 is considered statistically significant.

Based on the gene expression profile of UVM patients, GSEA was used to analyze the biological differences between groups. GSEA is a calculation method to analyze a statistical difference between two biological states in one gene set. GSEA is commonly used to estimate the pathway in the sample data set and the activity change of biological process [18]. The “c2.cp.kegg.v6.2.- symbols” gene set from MSigDB database for GSEA analysis, and adjusted P-value less than 0.05 is considered statistically significant. Trait genes in related pathways downloaded from GeneCards database were used to calculate each patient’s enrichment score in various pathways via the ssGSEA analysis algorithm.

### 2.4. Comparison on immune infiltration and immune related score

To quantify the proportion of different immune cells in UVM samples, we distinguished 22 human immune cell types in tumor microenvironment via the CIBERSORT method and LM22 gene set with high sensitivity and specificity, including B cells, T cells, natural killer cells, and macrophages, etc [19]. The CIBERSOFT method is a deconvolution algorithm to estimate the immune composition of various mixed cells from bulk tumor samples according to a reference gene set including 547 genes, which was regarded as the minimize representation of each cell type. Mann-Whitney U test was used on the comparison between immune infiltration of two groups.

The ESTIMATE method is an algorithm to quantify the immune activity (immune infiltration) of tumor samples based on the gene expression profile [20]. The R package ESTIMATE was applied to evaluate each tumor immune activity. Mann-Whitney U test was used on the comparison between immune infiltration of two groups.

### 2.5. Prognostic model construction and validation

Nomogram is widely used for cancer prognosis, mainly because of their ability to reduce statistical predictive models into a single numerical estimate of the probability. In order to further evaluate an influence of *CARD11* expression combined with clinicopathological characteristics on the prognosis of patients, the univariate and multivariate Cox analyses were used to analyze the independent prediction ability of risk score in combination with clinicopathological characteristics for OS. Harrell’s Consistency index was measured for the quantification of discriminant performance. Then calibration curve was generated by a comparison between the predicted values of the nomogram and the observed actual survival rates.

### 2.6. The PPI network construction and hub genes identification

Search Tool for the Retrieval of Interacting Genes (STRING, https://string-db.org/) database (v11.0), an online biological database designed for predicting protein-protein interaction (PPI) networks, was used to analyze an interactive relationship among DEGs. A combined score>0.4 was selected as significant. CytoHubba, a pluggable unit of Cytoscape (v3.7.2), was used to visualize the PPI network and calculate the Maximal Clique Centrality (MCC) [21, 22]. The eight genes with top highest MCC was identified as hub genes.

### 2.7. Establishment of ceRNA network

Before an analysis of basic statistical data, we downloaded the lncRNA-miRNA interaction information from the miRcode database (http://www.mircode.org). The miRNA-mRNA interaction information was downloaded from the miRTarBase database (http://miRTarBase.cuhk.edu.cn/), miRDB database (http://www.mirdb.org), and TargetScan database (http://www.targetscan.org/vert_72/). The “limma” package was used to analyze the differential miRNAs and lncRNAs between high- and low-expressed group. The log fold change (logFC) > 1.5 and the corrected *P* < 0.05 were regarded as significant. Subsequently, Cytoscape (v3.7.2) was performed to construct a ceRNA network based on a related analysis of miRNAs which were co-regulated by lncRNA and mRNA [21].

### 2.8. Statistical analysis

All data processing and statistical analysis was produced by using R (v 4.0.2). For the comparison of continuous variables between two groups, the statistical significance of normally distributed variables was estimated by the independent Student’s T-test. The difference between non-normal distributed variables was analyzed by the Mann-Whitney U test (namely Wilcoxon rank sum test). The Chi-square test or Fisher’s exact test was used to compare and analyze the statistical significance of categorical variables between two groups. The associated coefficients among different genes were calculated by Pearson correlation coefficient analysis. The survival package in R software was used for survival analysis. Kaplan-Meier curve was used to display the survival difference, and log-rank test was used to evaluate the significance of the difference in survival time between two groups. The univariate and multivariate Cox regression analyses were utilized to determine independent prognostic factors. The receiver operator characteristic (ROC) curve was drawn using the pROC package, and the area under curve was calculated to assess the accuracy of risk score in order to estimate the prognosis [23]. All statistical P-values were bilateral and P<0.05 was considered statistically significance.

## 3. Results

### 3.1. Correlation between CARD11 expression and clinical features

**Fig 1:**
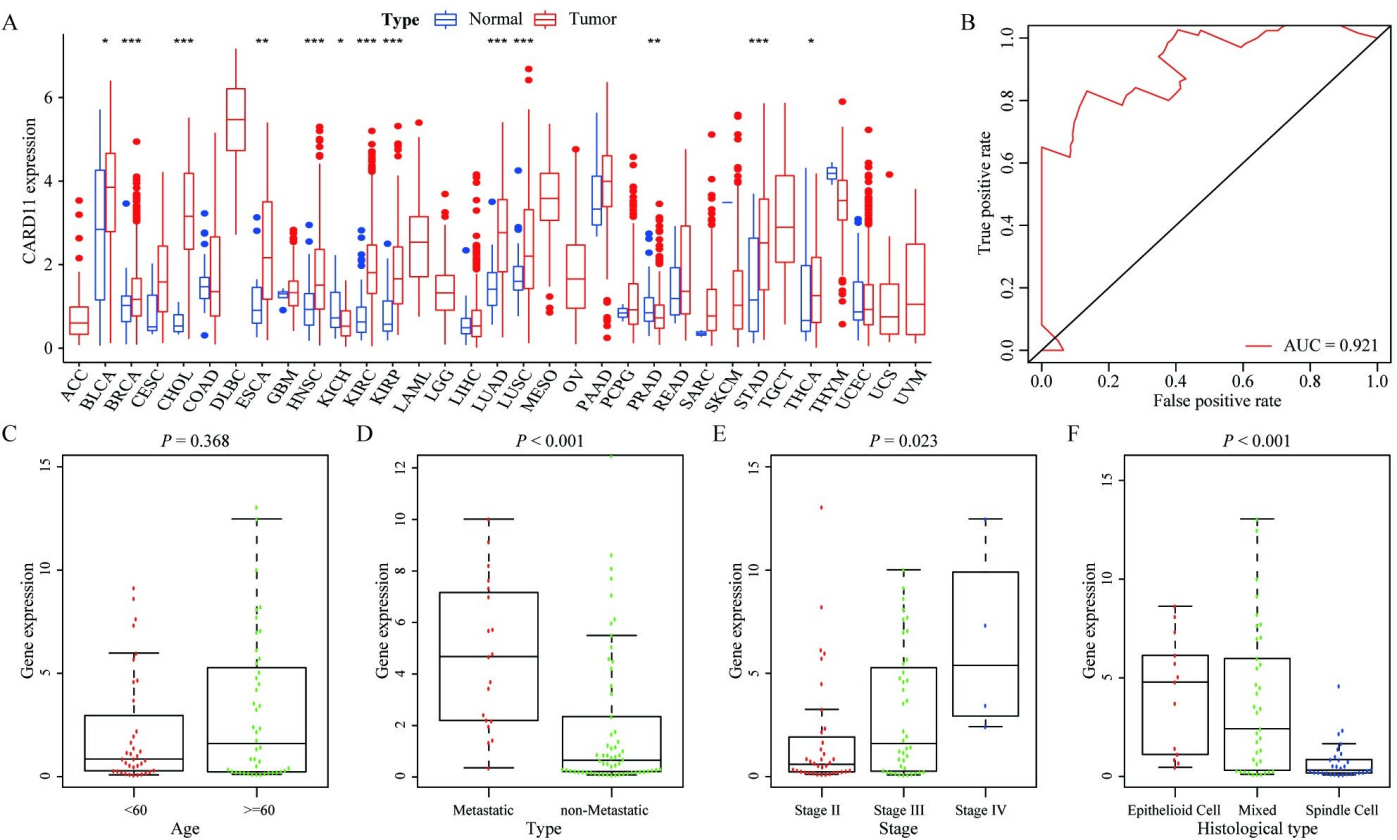
Correlation analysis between *CARD11* expression and clinicopathological characteristics. (**A**) Expression of *CARD11* in 33 types of tumors and their adjacent tissues in TCGA database; (**B**) ROC curve analysis of the accuracy of *CARD11* in predicting the prognosis of patients; (**C-F**) Correlation between *CARD11* expression and clinicopathological characteristics, in which *CARD11* have no significant correlation with age (*P* = 0.83; **C**), but have significant correlation with a higher likelihood of metastasis (*P* < 0.001; **D**), higher grade clinical stage (*P* = 0.023; **E**), and tissue cell typing (*P* < 0.001; **F**).

In the TCGA database, the expression of *CARD11* in most tumors was significantly higher than that in adjacent normal tissues (Fig 1A). Furthermore, ROC analysis showed that *CARD11* predicted the prognosis of UVM patients well (area under curve = 0.921; Fig 1B). The Kruskal–Wallis test and Wilcoxon rank test were performed to further analyze the correlation between *CARD11* expression and clinicopathological features in UVM patients. Results showed that the high *CARD11* expression was closely associated with higher possibility of metastasis (*P* < 0.001; Fig 1D), higher grade clinical stage (*P* = 0.023; Fig 1E) and histiocytic classification (*P* = 0.368; Fig 1F), but was not significantly associated with age (*P* = 0.368; Fig 1C).

### 3.2. Prognostic value of CARD11 expression

**Fig 2:**
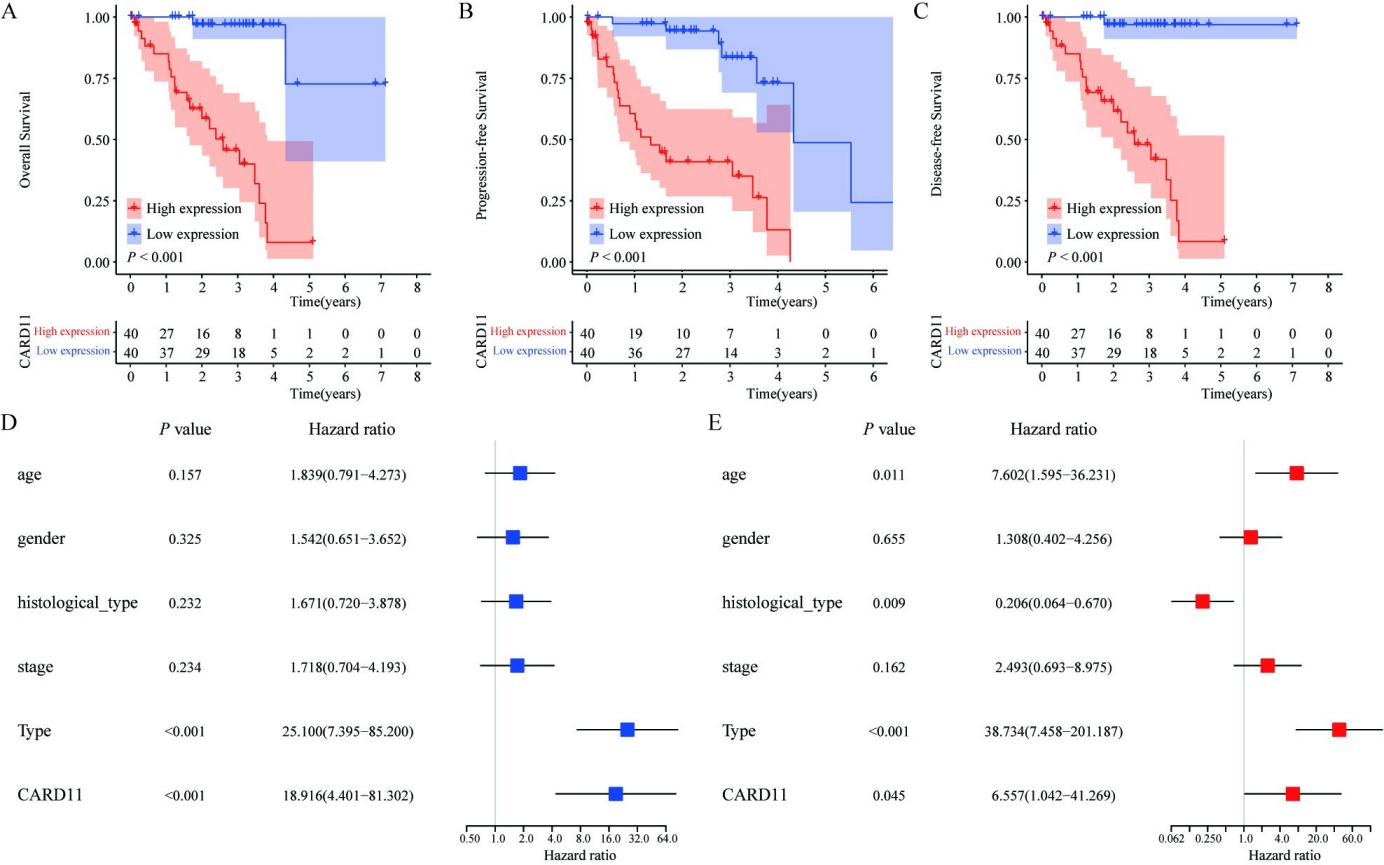
Influence of *CARD11* expression on the prognosis of UVM patients. (**A-C**) Kaplan-Meier analysis showed that the OS, PFS, and DFS in *CARD11* high-expression group were lower (log-rank *P* <0.001), comparing with that in *CARD11* low-expression group. (**D, E**) The univariate and multivariate Cox regression analysis combined *CARD11* expression with clinicopathological characteristics showed that *CARD11* was an independent risk factor for prognosis.

We then analyzed the correlation between the expression of *CARD11* and the prognosis outcome of OS, Progression-free Survival (PFS), and Disease-free Survival (DFS). The high expressed group was associated with poor OS (Log-rank *P* < 0.001, Fig 2A), PFS (Log-rank *P* < 0.001, Fig 2B) and DFS (Log-rank *P* < 0.001, Fig 2C). In addition, univariate and multivariate Cox regression analyses were used to evaluate OS (Fig 2D and 2E) and concluded that *CARD11* expression is an independent predictor for OS [HR = 6.557 (1.042–41.269), *P* = 0.045, Table 2].

**Table 2:** Univariate and multivariate Cox regression analyses for overall survival of uveal melanoma patients based on CARD11 expression.

Variables	Univariate Cox analysis		Multivariate Cox analysis	
	HR (95% CI) *P value*		HR (95% CI)	*P value*
Age (≥60 vs. <60)	1.83(0.79–4.27)	0.157	7.60(1.60–36.23)	0.011*
Gender (male vs. female)	1.54(0.65–3.65)	0.325	1.31(0.40–4.26)	0.655
Histological type (Mixed vs. Others)	1.67(0.72–3.88)	0.232	0.21(0.06–0.67)	0.008**
Stage (III+IV vs. I+II)	1.72(0.70–4.19)	0.234	2.49(0.69–8.97)	0.162
Type (metastatic vs. non- metastatic)	25.10(7.39–85.20)	<0.001***	38.73(7.46–201.19)	<0.001***
CARD11 (high vs. low)	18.92(4.40–81.30)	<0.001***	6.56(1.04–41.27)	0.045*

### 3.3. Correlation between CARD11 expression and different biological characteristics

**Fig 3:**
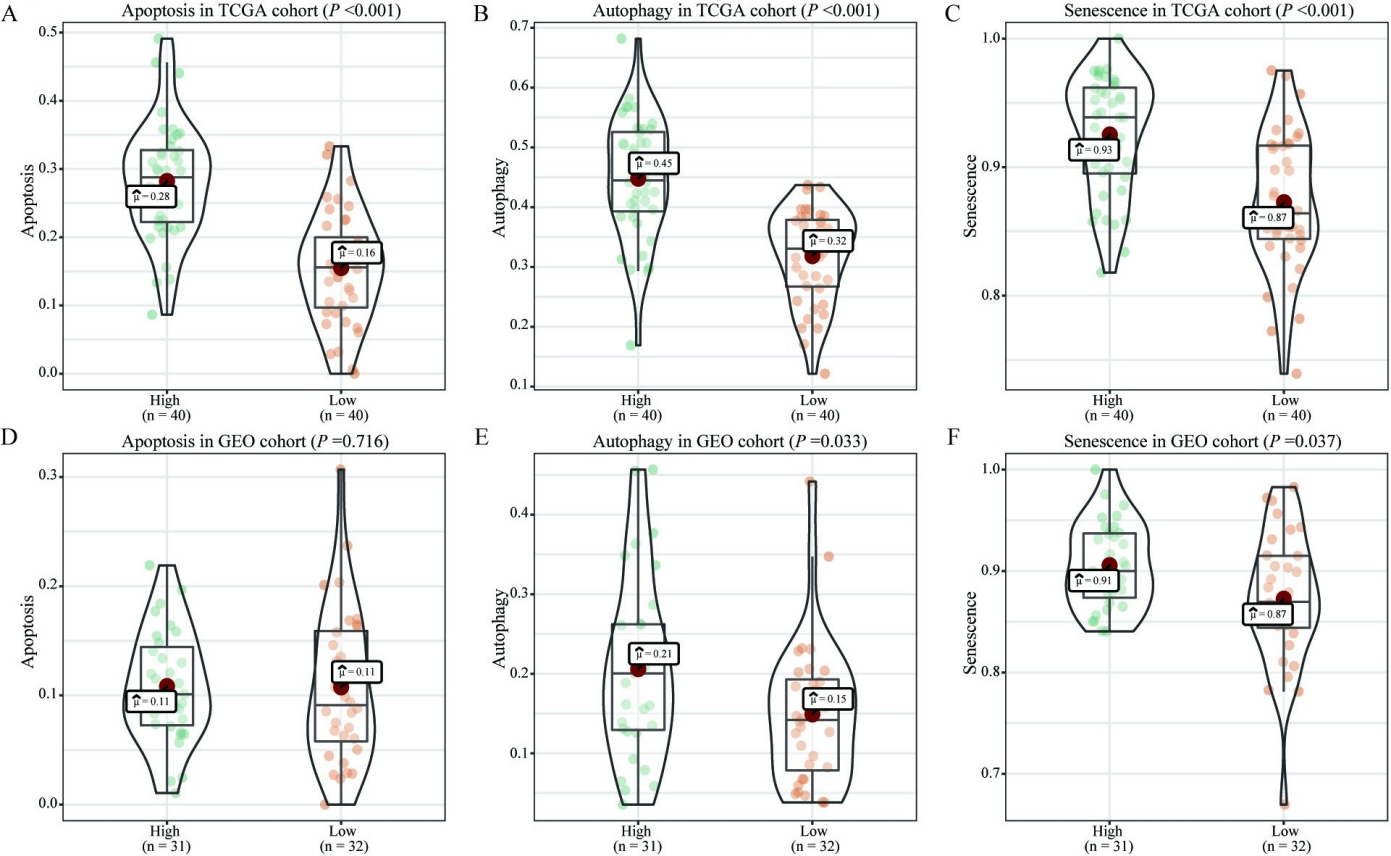
Association between *CARD11* expression and different biologically related pathways. (**A-C**) Correlation analysis showed that in TCGA database, patients with high *CARD11* expression had higher scores of apoptosis, autophagy, and cell senescence related pathways. (**D-F**) In GEO database, patients with high *CARD11* expression had higher autophagy and senescence related pathway scores, while there was no significant difference between the two groups in apoptosis related pathway scores.

We further analyzed the effects of *CARD11* expression on different biological phenomenon. The results indicated that patients with high *CARD11* expression had markedly higher apoptosis related scores (*P* < 0.001; Fig 3A), autophagy related scores (*P* < 0.001; Fig 3B), and cellular senescence related scores (*P* < 0.001; Fig 3C) in the TCGA database. GSE22138 also indicated that high *CARD11* expression had closely positive correlation to autophagy (*P* = 0.037; Fig 3E) and cellular senescence related scores (*P* = 0.037; Fig 3F), with no significant relativity to apoptosis. (*P* = 0.716; Fig 3D)

### 3.4. The correlation between CARD11 expression and immune infiltration

**Fig 4:**
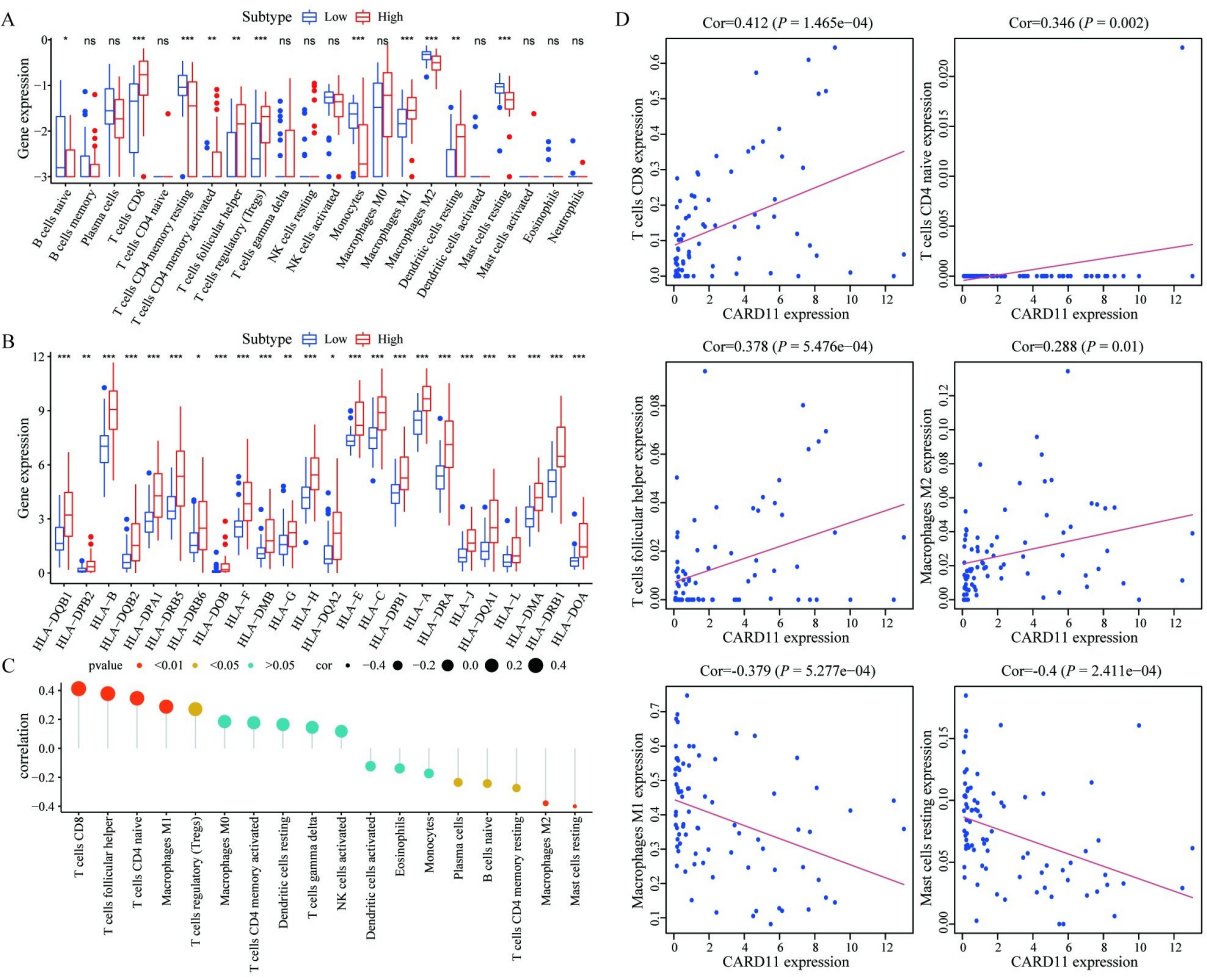
The correlation between *CARD11* expression and immune infiltration. (**A**) Correlation analysis showed that the infiltration level of some immune cell subtypes was significantly different between the high and low expression groups. (**B**) The expression levels of HLAs were significantly different between the high and low expression groups. (**C**) Lollipop graph showed that the correlation between different immune cell subtypes and *CARD11* expression. The size of the dots represents the correlation coefficient, and the color represents different levels of P values. (**D**) Analysis of immune cell subtypes significantly related with *CARD11* expression.

We estimated the association of *CARD11* expression with 22 different immune cell subsets and Human Leukocyte Antigen (HLA) gene families in TCGA UVM patients. The results showed that the infiltration level of some immune cell subsets was significantly different between the high- and low-*CARD11* expression groups. Infiltration level of B cell naïve, T cell CD4 memory resting, Monocytes, Macrophages M2, Mast cells resting was higher in *CARD11* low-expression group. Infiltration level of T cells CD8, T cells CD4 memory activated, T cells follicular helper, T cells regulatory (Tregs), Macrophages M1, Dendritic cells resting was lower in *CARD11* low-expression group (Fig 4A). At the meanwhile, the expression level of HLAs was significantly positively correlated with high *CARD11* expression (Fig 4B). Subsequently, we calculated the correlation of *CARD11* expression with 22 different immune cell subsets and HLA gene families, and found that *CARD11* expression had significant and positive correlation with T cells CD8+, T cells CD4+ naive, T cells follicular helper, Macrophages M1 and significantly negative correlation with Macrophages M2 and Mast cells resting (Fig 4C and 4D).

### 3.5. Nomogram construction for prediction of OS

**Fig 5:**
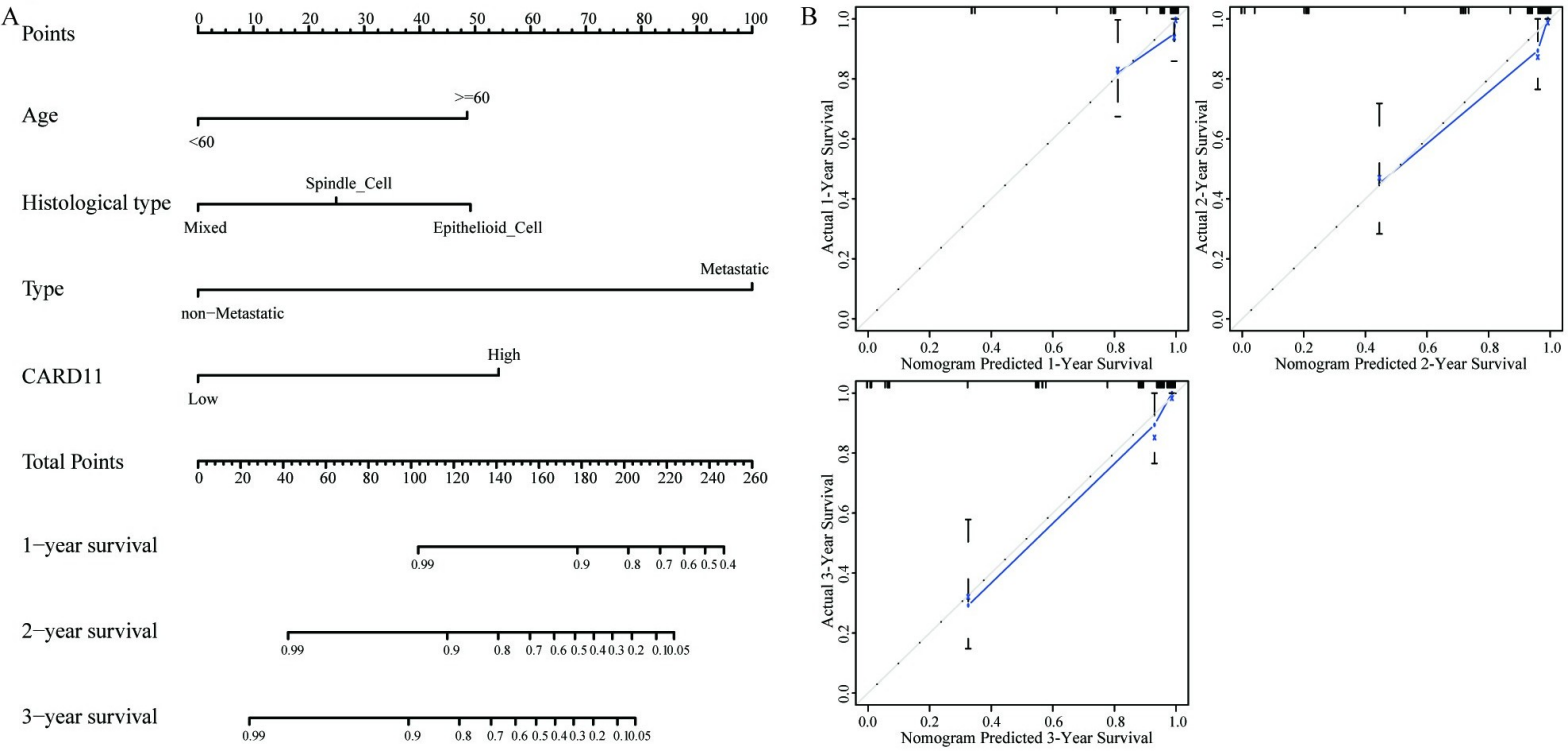
Nomogram construction for survival prediction. (**A**) Nomogram construction combined *CARD11* expression with clinicopathological features to predict the OS of patients. (**B**) Calibration curve of Nomogram; the Abscissa is the survival predicted through the nomogram, and the ordinate is the actual observations, repeating 1000 times each time. The curve showed that the nomogram predicted the 1-, 2-, and 3-year OS in UVM patients effectively.

We constructed a nomogram which combined *CARD11* expression with clinicopathological characteristics to predict the OS of UVM patients, and Consistency index showed a high distinct degree [0.919 (0.872–0.966)] of the nomogram (Fig 5A). Besides, we used a calibration plot to verify the prediction efficiency of the model. The 1-, 2-, and 3-year OS in nomograms were accordant with the values of actual patient’s observations, which suggested that the OS prognosis model effectively predicted the 1-, 2-, and 3-year OS in UVM patients (Fig 5B).

### 3.6. The relationship between CARD11 expression and entire gene expression profile

To further analyze the relationship between *CARD11* expression and entire gene expression profile in UVM patients, we divided patients into high *CARD11* expression group and low *CARD11* expression group and analyzed DEGs between the two groups.

**Fig 6:**
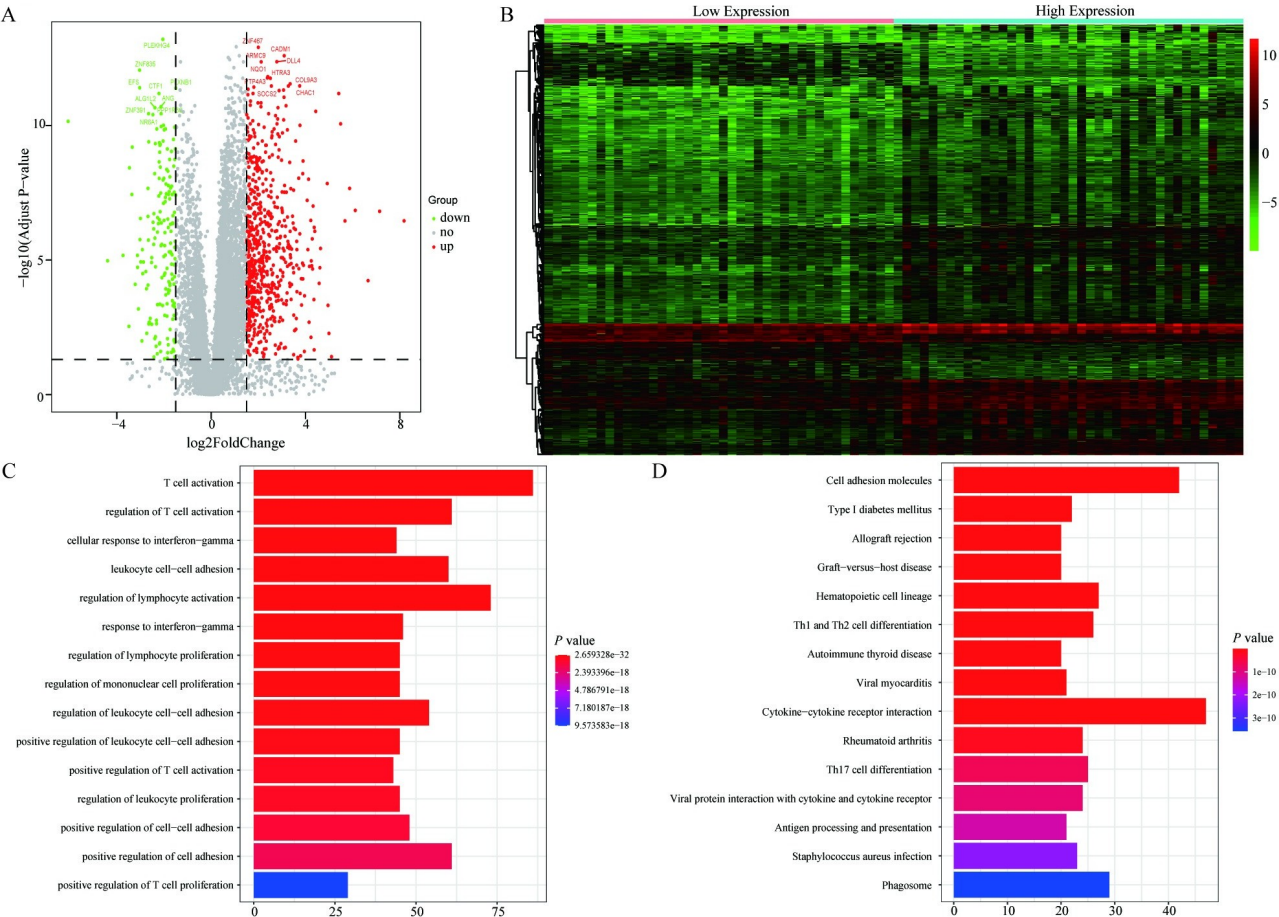
Analysis of differential genes between *CARD11* high and low expression groups. (**A, B**) Heat maps and volcano plots showed the expression of DEGs between *CARD11* high and low expression group. (**C, D**) GO and KEGG analysis showed that these DEGs were involved in T cell activation pathway and cell adhesion molecule-related signal pathway.

**Fig 7:**
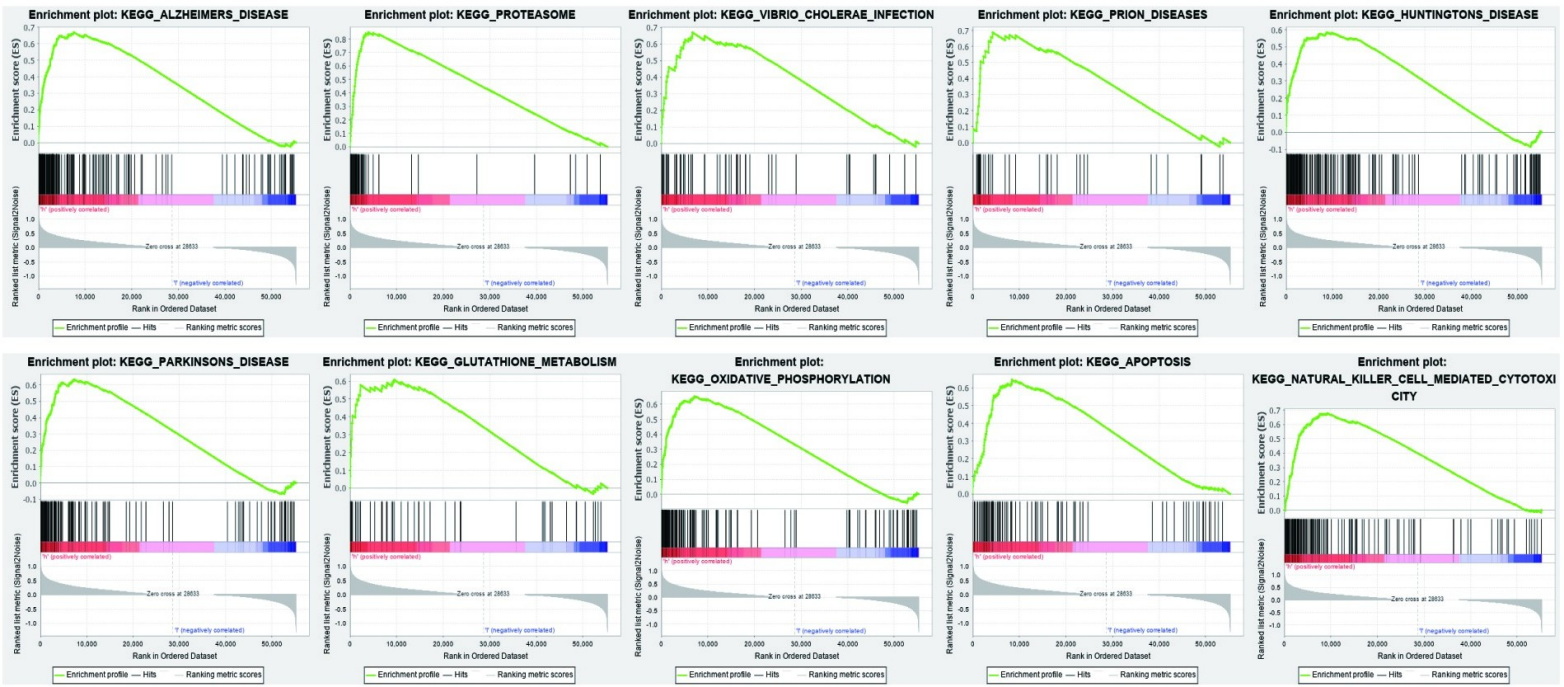
The GSEA analysis based on *CARD11* expression. The results revealed that some pathways were enriched in UVM patients with *CARD11* high expression, including Alzheimer’s Disease, Proteasome, Vibrio cholerae infection, and Prion Diseases pathways.

The results revealed that 860 DEGs were identified as being statistically significant (|Log_2_FC | > 1.5 and FDR < 0.05) (S1 Table), and heatmaps and volcano plots were shown in Fig 6A. Subsequently, we performed GO and KEGG analysis of DEGs, and found that DEGs were closely related to T cell activation pathways and cell adhesion molecules (Fig 6C and 6D). Meanwhile, GSEA results showed that pathways involved in Alzheimer’s Disease, Proteasome, Vibrio Cholerae infection and Prion Diseases were significantly enriched in patients with high *CARD11* expression (Fig 7, Table 3).

**Table 3:** GSEA analysis.

Name	Size	Enrichment Score	NES	FDR	Leading edge
KEGG_ALZHEIMERS_DISEASE	46165	0.67	1.88	0.010	tags = 58%, list = 14%, signal = 67%
KEGG_PROTEASOME	46	085	1.83	0.036	tags = 78%, list = 7%, signal = 84%
KEGG_VIBRIO_CHOLERAE_INFECTION	54	0.67	1.81	0.013	tags = 48%, list = 12%, signal = 55%
KEGG_PRION_DISEASES	35	0.69	1.79	0.014	tags = 46%, list = 8%, signal = 49%
KEGG_HUNTINGTONS_DISEASE	180	0.58	1.78	0.016	tags = 52%, list = 16%, signal = 61%
KEGG_PARKINSONS_DISEASE	127	0.63	1.76	0.017	tags = 55%, list = 13%, signal = 63%
KEGG_GLUTATHIONE_METABOLISM	49	0.61	1.74	0.034	tags = 47%, list = 17%, signal = 57%
KEGG_OXIDATIVE_PHOSPHORYLATION	131	0.65	1.74	0.032	tags = 56%, list = 13%, signal = 65%
KEGG_APOPTOSIS	87	0.65	1.71	0.037	tags = 54%, list = 15%, signal = 64%
KEGG_NATURAL_KILLER_CELL_MEDIATED_CYTOTOXICITY	132	0.68	1.68	0.054	tags = 59%, list = 17%, signal = 71%

### 3.7. Construction of PPI and ceRNA networks

**Fig 8:**
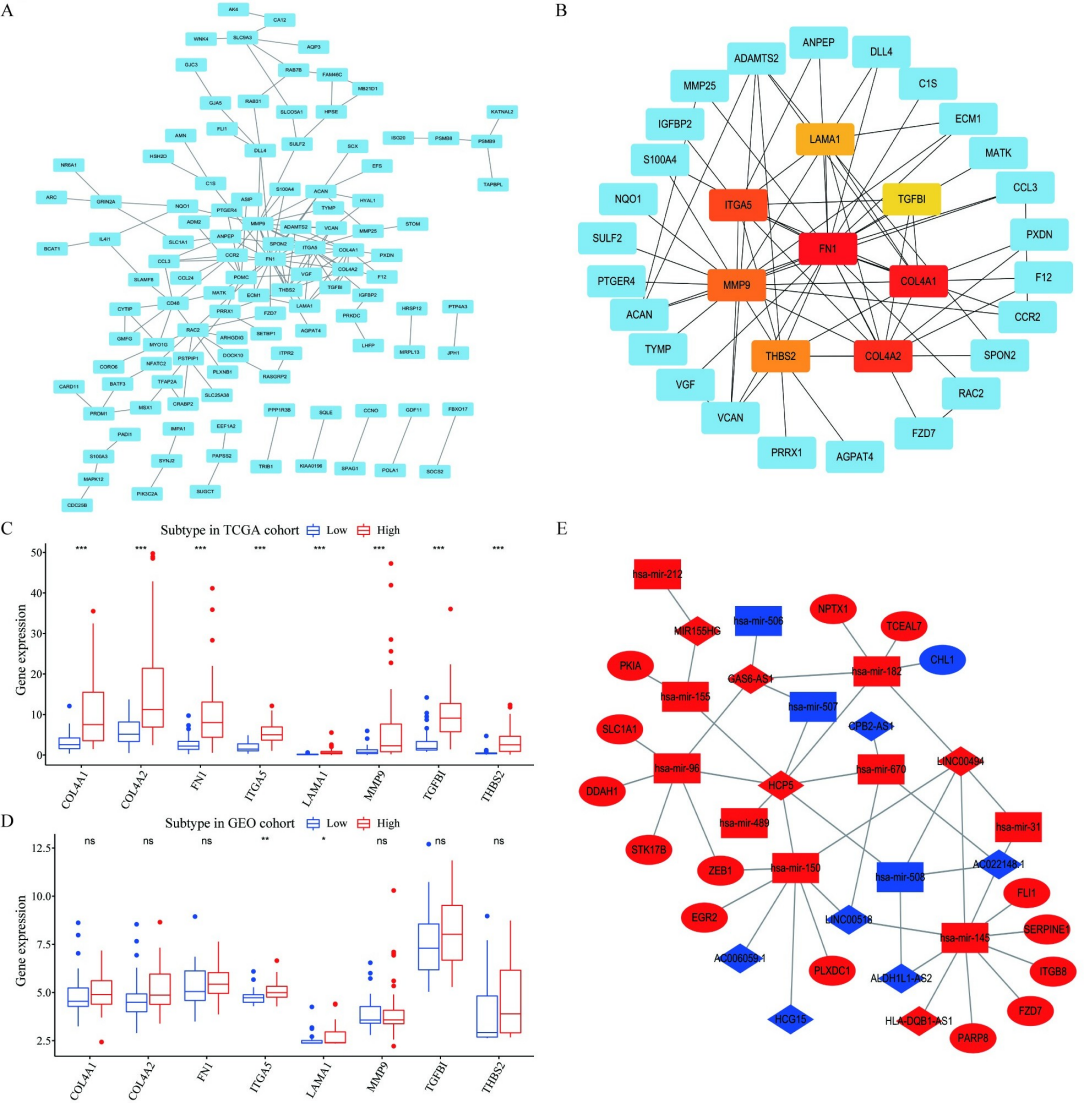
PPI network and visualization of ceRNA network. (**A**) STRING database was used to analyze the PPI network of 193 prognostic genes, and each node represented a distinct gene. (**B**) MCC algorithm was used to recognize hub-genes from PPI network, red and yellow nodes represented 8 hub-genes; (**C, D**) The expression of 8 hub-genes in the TCGA database and GEO database; (**E**) The ceRNA network was built based on differentially expressed mRNA, miRNA, and IncRNA. Among them, ellipse represented mRNA, diamond represented IncRNA, and square represented miRNA. Red suggests elevated gene expression and blue suggests decreased gene expression.

Total of 193 genes (*P* < 0.001) associated with prognosis were screened out from the 860 differential genes by using the univariate Cox regression analysis (S2 Table). STRING database was used to build a predicting PPI network among genes (Fig 8A), and eight genes with the highest MCC were selected out as hub genes by using Cytoscape plug-in CytoHubba (Fig 8B). Meanwhile, the differential analyses showed that the expressions of hub genes all increased in the high *CARD11* expression group in the both TCGA and GEO databases (Fig 8C and 8D). The ceRNA network was built based on differentially expressed mRNA, miRNA, and lncRNA (Fig 8E, S3 and S4 Tables).

## 4. Discussion

UVM is the most common primary intraocular malignancy, which is associated with high mortality because of a high propensity to metastasize [24]. The common clinical therapies, such as systemic chemotherapy, monotherapy, and combination therapy, cannot prolong OS in UVM patients [25]. Therefore, it is urgent to find the feasible therapeutic targets and effective treatment. *CARD11*, whose mutation was well known as the cause of severe T- and B-cell immune deficiency, has been reported could promote human diffuse large B cell lymphoma and some other tumorigenesis [26, 27]. However, the correlation between *CARD11* and UVM is unclear. In this study, we analyzed prognostic value and immune-related characteristics of *CARD11* with a construction of ceRNA network in UVM, hoping to provide a potential management and research direction.

Previous studies showed that *CARD11* somatic mutations were detected frequently in multiple human cancer types, including lymphoma, colorectal cancer, and triple-negative breast cancer [28–31]. In our study, we demonstrated that the mRNA expression level of *CARD11* was elevated in UVM tissues via TCGA database. Based on univariate and multivariate Cox regression analyses and a nomogram, our results showed that *CARD11* was significantly associated with worse clinicopathological parameters, such as shorter OS, PFS and DFS. We also found that high expression of *CARD11* was positively correlated with apoptosis and cellular senescence, which were universal and important during the development of tumor, indicating *CARD11* might influence the prognosis of UVM patients by regulating autophagy and senescence of tumor cells. Frequent *CARD11* mutations were detected in diffuse large B cell lymphoma [27]. Some literature indicated *CARD11* had prognostic value in diffuse large B cell lymphoma [32, 33]. Considering its overarching value in UVM, its development is welcome where the prognostication needs to be improved and inhibition of *CARD11* expression may have potential significance for improving UVM prognosis.

*CARD11*, mainly expressed in lymphoid tissues, is reported to be associated with immune cells and plays an important role in carcinogenesis [7, 34]. In order to analyze the correlation between *CARD11* expression and immune infiltrations in UVM, we assessed the immune cell infiltration levels between the two groups. These results showed that there were significant differences in the infiltration levels of some immune cell subsets between the high- and low-*CARD11* groups. Besides, *CARD11* expression had a significantly positive correlation with T cells CD8+, T cells CD4+ naïve, and T cells follicular helper, which indicated that *CARD11* expression was closely related to T cells subtypes, and had a closely negative correlation with mast cells, which played a protective role in tumor, but also had the ability to promote angiogenesis and tumor progression [35]. Interestingly, we found that *CARD11* expression was positively correlated with Macrophages M2, which was capable of promoting tumor progression, and negatively correlated with Macrophages M1, which had an anti-tumor capability [36–38]. As we know, *CARD11* is involved in the signal transduction of T cells by regulating NF-κB activation in immune response. *CARD11-BCL10-MALT1* signaling complex can regulate lymphocyte proliferation, differentiation, and survival [5, 6]. Tumor immune microenvironment (TIME) is also closely related to angiogenesis, which is a key event in the occurrence and development of UVM [39]. Xue Xu et al. divided cancer patients into different TIME subtypes and found that patients with TIME-rich tumor had better prognosis than patients with TIME-poor and TIME-intermediate tumor, indicating that *CARD11* might affect the prognosis of UVM patients by participating in the regulation of TIME [40]. In summary, our results implied the crucial role of *CARD11* expression and immune cell infiltrations in UVM development, and also provided a potential research direction for immunotherapy.

In this study, hub genes identified by Cytohubba were related with tumor invasion and metastasis or tumor suppression. *FN1*, *ITGA5*, *THBS2*, and *LAMA1* were associated with cell adhesion and metastasis, and the expression of *TGFB1*, *COL4A1*, *COL4A2*, and *THBS2* inhibited tumor growth. Some studies suggested *MMP9* played a role in modulateing neovessel remodeling and promoted tumor growth [41]. These also accords with our earlier analysis, which showed that high *CARD11* expression was correlated with possibility of metastasis. These can be further evidence of *CARD11* playing a momentous role in UVM progression via a multiple regulation of tumor invasion, metastasis, and tumor microenvironment.

CeRNAs refer to transcripts that can regulate each other at post-transcription level by competing for shared miRNAs [42]. Previous study has demonstrated that the ceRNA activity was determined by factors may lead to ceRNA network imbalance and thus contribute to cancer initiation and progression, such as miRNA/ceRNA abundance, ceRNAs binding affinity to miRNAs, RNA editing, and RNA-binding proteins. It was also revealed that ceRNAs involved in the pathogenesis of several common cancers such as prostate cancer, liver cancer, breast cancer, lung cancer, gastric cancer, and endometrial cancer [43]. In this study, we constructed a ceRNA network with differential expressed mRNA, miRNA, and lncRNA. The ceRNA mechanism facilitated a large-scale regulatory network cellular activity [44]. We can get more details about the impact of *CARD11* expression on UVM by studying the ceRNA network. Many genes of this network are related to various cancers. *HPC5* is a genetic locus associated with prostate cancer, and *GAS6-AS1* is associated with lung cancer, renal cell carcinoma, and papillary. *MIR155HG* is correlated with high grade glioma, leukemia, and chronic lymphocytic, and *STK17B* is related to colon squamous cell carcinoma. Furthermore, *FLI1* is related to transcriptional misregulation in cancer and NF-κB signaling pathway. *ZEB1* promotes tumorigenicity by repressing stemness-inhibiting microRNAs. The ceRNA findings we put at the last part of our research was to provide novel evidence that *CARD11* plays an important role in tumorigenesis, which would help further our understanding of the mechanism underlying the pathogenesis of UVM.

However, there are several limitations to the present study. Firstly, only *CARD11* mRNA expression levels were examined as a potential prognostic biomarker to predict OS, PFS and DFS times, so further validation studies should be analyzed to verify the present findings. Secondly, despite conducting bioinformatics analysis of functional annotations and enrichment of *CARD11*-associated pathways, these findings were not verified by exploring the underlying molecular mechanisms of *CARD11* signaling. Thus, further studies with more varied samples will be required to understand the association between *CARD11* and tumor growth in UVM, as well as in other cancer types.

## 5. Conclusions

In summary, the study revealed the prognostic value of *CARD11* in UVM. Our data demonstrated that the high *CARD11* expression in UVM is associated with poor OS, PFS and DFS, indicating that *CARD11* may serve as a potential biomarker for the diagnosis and prognosis of the UVM. Further experimental validation should be performed to prove the biologic impact of *CARD11* on UVM.

## Supporting information

S1 Table DEGs between high and low CARD11 expression groups. (XLS)

S2 Table Univariate Cox analysis of 860 DEGs. (XLS)

S3 Table Differentially expressed miRNA between high and low CARD11 expression groups. (XLS)

S4 Table Differentially expressed lncRNA between high and low CARD11 expression groups. (XLS)
